# Effect of face masks on speech perception in noise of individuals with hearing aids

**DOI:** 10.3389/fnins.2022.1036767

**Published:** 2022-12-01

**Authors:** Jung Ho Choi, Hyo Jung Choi, Dong Hyun Kim, Ji Hye Park, Yong-Hwi An, Hyun Joon Shim

**Affiliations:** Department of Otorhinolaryngology-Head and Neck Surgery, Nowon Eulji Medical Center, Eulji University School of Medicine, Seoul, South Korea

**Keywords:** face mask, pandemic, speech perception, noise, hearing impairment, hearing aid

## Abstract

Although several previous studies have confirmed that listeners find it difficult to perceive the speech of face-mask-wearing speakers, there has been little research into how masks affect hearing-impaired individuals using hearing aids. Therefore, the aim of this study was to compare the effects of masks on the speech perception in noise of hearing-impaired individuals and normal-hearing individuals. We also investigated the effect of masks on the gain conferred by hearing aids. The hearing-impaired group included 24 listeners (age: *M* = 69.5, *SD* = 8.6; M:F = 13:11) who had used hearing aids in everyday life for >1 month (*M* = 20.7, *SD* = 24.0) and the normal-hearing group included 26 listeners (age: *M* = 57.9, *SD* = 11.1; M:F = 13:13). Speech perception in noise was measured under no mask–auditory-only (no-mask–AO), no mask–auditory–visual (no-mask–AV), and mask–AV conditions at five signal-to-noise ratios (SNRs; −16, −12, −8, −4, 0 dB) using five lists of 25 monosyllabic Korean words. Video clips that included a female speaker’s face and sound or the sound only were presented through a monitor and a loudspeaker located 1 m in front of the listener in a sound-attenuating booth. The degree of deterioration in speech perception caused by the mask (no-mask–AV minus mask–AV) was significantly greater for hearing-impaired vs. normal-hearing participants only at 0 dB SNR (Bonferroni’s corrected *p* < 0.01). When the effects of a mask on speech perception, with and without hearing aids, were compared in the hearing-impaired group, the degree of deterioration in speech perception caused by the mask was significantly reduced by the hearing aids compared with that without hearing aids at 0 and −4 dB SNR (Bonferroni’s corrected *p* < 0.01). The improvement conferred by hearing aids (unaided speech perception score minus aided speech perception score) was significantly greater at 0 and −4 dB SNR than at −16 dB SNR in the mask–AV group (Bonferroni’s corrected *p* < 0.01). These results demonstrate that hearing aids still improve speech perception when the speaker is masked, and that hearing aids partly offset the effect of a mask at relatively low noise levels.

## Introduction

As COVID-19 infections have spread worldwide, mask-wearing is recommended for preventing infection and transmission. However, the widespread use of masks causes inconvenience in everyday conversation. Many previous studies have confirmed that mask-wearing interferes with communication not only in hearing-impaired individuals, but also in individuals with normal hearing (Atcherson et al., 2017; Chodosh et al., 2020; Helfer et al., 2021; Rahne et al., 2021; Thibodeau et al., 2021). Listeners recalled significantly fewer words when the sentences were spoken with a face mask, because when speech delivered with a face mask is processed, fewer cognitive resources are available for storing speech in memory (Truong and Weber, 2021). Furthermore, the ability to perceive masked speech does not improve with increasing experience of listening to mask-wearers (Crinnion et al., 2022). A large-scale survey reported that all respondents had trouble understanding masked speech, regardless of their age and hearing status, and that increased concentration was required to understand speech when communicating in noise with someone wearing a face mask (Helfer et al., 2021). Hearing-impaired patients attending outpatient clinics complain of more discomfort in communicating with healthcare providers than they experienced before the pandemic. This is an emerging social problem because it can significantly affect the accurate diagnosis and treatment of illness.

The adverse effects of masks on speech perception can be explained by the consequent sound attenuation (Corey et al., 2020; Magee et al., 2020; Rahne et al., 2021) and the elimination of visual cues derived from the motion of the speaker’s lips or mouth (Atcherson et al., 2017; Bernstein et al., 2020; Thibodeau et al., 2021). A recent study reported that participants who had difficulty in perceiving masked speech complained of sound attenuation (44.1%) and the impossibility of lip reading (55.9%) (Trecca et al., 2020). Many previous studies have confirmed that most kinds of mask attenuate sound at frequencies above 1 or 2 kHz (Corey et al., 2020; Magee et al., 2020; Rahne et al., 2021), so that the perception of masked sound is similar to the perception of sound by a person with high-frequency hearing loss. High-frequency sound attenuation decreases spectral resolution, making it particularly difficult to discriminate words with high-pitched consonants (e.g., /t/,/d/,/k/,/z/, and/s/). Another effect of a mask on speech perception is that it blocks the visual information imparted by the speaker’s mouth. Poorer speech perception under the auditory-only (AO) condition than under the auditory-visual (AV) condition is entirely predictable (O’Neill, 1954). The visual contribution to speech perception is known to be more important under noisy conditions (O’Neill, 1954; Sumby and Pollack, 1954). As the environmental noise level increases, speech perception is more strongly compromised under the AO condition than under the AV condition (Sumby and Pollack, 1954), although several studies have reported that the gain in speech perception with visual articulation is maximal at a signal-to-noise ratio (SNR) of −12 dB (Ross et al., 2007; Liu et al., 2013). Because the visual cues provided by the lip shape and tongue position best complement the perception of high-frequency voice signals (MacLeod and Summerfield, 1990), the concealment of mouth movements must be disastrous for the recognition of masked speech, which is attenuated above 2 kHz (Corey et al., 2020; Magee et al., 2020; Rahne et al., 2021).

Hearing-impaired listeners may have greater difficulty perceiving masked speech, because they are more susceptible than normal-hearing individuals to the sound attenuation caused by the mask and depend more strongly on lip reading during conversation. Recent surveys have shown that hearing-impaired listeners were significantly more affected by masked speech than those without hearing impairment (Saunders et al., 2021; Poon and Jenstad, 2022). Two behavioral studies reported that the adverse effect of masks on speech perception is greater in hearing-impaired individuals than in normal-hearing individuals (Thibodeau et al., 2021; Alkharabsheh et al., 2022). The study by Thibodeau et al. used single levels of speech and noise presentation at −5 dB SNR, and the study by Alkharabsheh et al. used relatively robust signals (quiet, + 10, + 5, and 0 dB SNR). Therefore, a comparison of hearing-impaired individuals and normal-hearing individuals at a wider range of noise levels is required. The hearing aid is the device most frequently used to assist individuals with hearing impairment, and the number of patients prescribed hearing aids increased significantly during the COVID-19 pandemic (Ertugrul and Soylemez, 2021). Although manufacturers are quickly developing programs to enhance the perception of high frequencies with their hearing aids to compensate for the acoustic effects caused by wearing masks (Bhowmik et al., 2021), most existing hearing aids and fitting programs are still developed to suit the daily conventions prior to the pandemic. Therefore, it is important and urgent to evaluate how much masked speech affects the gain conferred by existing hearing aids, to prepare for an environment where mask wearing continues for the foreseeable future.

We hypothesized that the effects of masks on hearing-impaired individuals are greater than those on individuals with normal hearing. Our second hypothesis was that masks reduce the benefit of hearing aids in speech perception, although hearing aids can partially compensate for the deterioration in speech perception caused by masks. To test our hypotheses, we compared the effects of masks on speech perception in hearing-impaired individuals and normal-hearing individuals against relatively intense background noise. We also investigated the effect of masks on speech perception in within-subject comparisons, by comparing the deterioration in speech perception caused by the mask in hearing-impaired individuals with and without hearing aids.

## Subjects and methods

### Subjects

**FIGURE 1:**
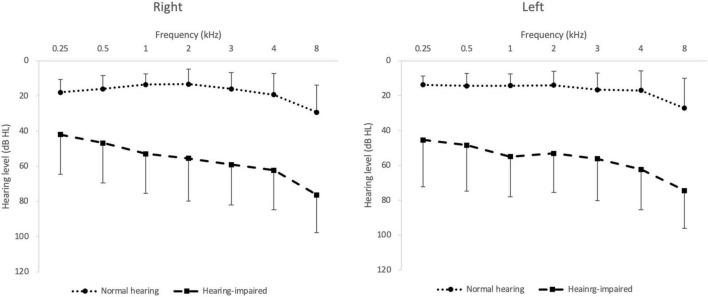
Mean pure-tone hearing thresholds in the normal-hearing (filled circles) and hearing-impaired group (filled squares). The error bar indicates SD.

All subjects spoke Korean as their native language. The hearing-impaired group included 24 elderly listeners (age: *M* = 69.5, *SD* = 8.6; M:F = 13:11) who had used hearing aids in everyday life for more than 1 month (duration: *M* = 20.7, *SD* = 24.0) and the normal-hearing group included 26 listeners (age: *M* = 57.9, *SD* = 11.1; M:F = 13:13). Table 1 shows the mean age, sex and pure-tone-average thresholds of the hearing-impaired group and detailed characteristics of their hearing aids. There was no significant difference between the two groups in the sex distribution (*p* > 0.05) but there was a significant difference in age (*p* < 0.05). The pure-tone averages across 500, 1,000, 2,000, and 3,000 Hz in the normal-hearing group and were 14.7 (*SD* = 6.8) dB hearing level (HL) on the right side and 14.8 (*SD* = 6.3) dB HL on the left side. In the hearing-impaired group, pure-tone averages were 53.5 (*SD* = 21.9) dB HL in the right ear and 53.2 (*SD* = 22.0) dB HL in the left ear (Figure 1). They used Phonak Audeo or Virto hearing aids (Sonova AG, Stäfa, Switzerland). All hearing aids were fitted with NAL–NL2, and the Audioscan Verifit 2 real-ear measurement system (Etymotic Design, Inc., Dorchester, ON, Canada) was used to confirm that the output levels of the hearing aids were in the target range. The manufacturer’s fitting software (Phonak Target) was used, with the adaptive parameters for noise reduction and the directional microphone set to the default values, as recommended by the fitting software. The subjects had just been fitted with hearing aids for the first time and had undergone more than two further fittings within 1 month after they started to use their hearing aids.

**TABLE 1:** Age, sex, hearing thresholds, and characters of hearing aids in the hearing-impaired group.

Subject No.	Age (years)	Sex	Pure tone average (right, dB HL)	Pure tone average (left, dB HL)	Location	Hearing aid type	Number of channels	Duration of hearing aid use (months)
1	69	M	68.8	62.5	Both	RIC	12	34
2	77	M	51.3	60.0	Both	ITC	8	52
3	69	M	60.0	52.5	Both	RIC	8	64
4	76	M	40.0	48.8	Both	RIC	8	4
5	64	F	8.8	56.3	Left	RIC	20	1
6	76	M	97.5	66.3	Left	ITC	8	72
7	77	M	59.0	120.0	Right	RIC	8	8
8	72	M	63.8	56.3	Both	RIC	12	14
9	63	F	120.0	48.8	Left	RIC	12	35
10	67	F	50.0	56.3	Both	RIC	8	48
11	76	M	51.3	75.0	Both	ITC	8	50
12	70	F	38.8	48.8	Both	RIC	12	1
13	71	M	40.0	23.8	Right	RIC	16	4
14	45	F	30.0	40.0	Left	CIC	12	6
15	76	M	33.8	53.8	Both	RIC	20	10
16	79	M	62.5	92.5	Right	ITC	20	1
17	71	F	43.8	27.5	Right	RIC	12	5
18	80	M	61.3	51.3	Right	RIC	12	2
19	65	M	53.8	56.3	Both	RIC	16	1
20	75	F	53.8	36.3	Both	RIC	16	2
21	62	F	42.5	46.3	Both	RIC	12	11
22	71	F	46.3	48.8	Both	RIC	12	1
23	68	F	65.0	8.8	Both	RIC	12	60
24	49	F	42.5	40.0	Both	RIC	16	10
Mean	69.5		53.5	53.2				20.7
SD	8.6		21.9	22.0				24.0

M, male; F, female; ITC, In-the-Canal; CIC, Completely-in-the-Canal; RIC, Receiver-in-Canal.

The study was conducted in accordance with the Declaration of Helsinki and the recommendations of the Institutional Review Board of Eulji Medical Center. Written informed consent was obtained from all subjects. After subjects signed the consent form, a copy was given to them.

### Monosyllabic speech perception test in noise

A female speaker recorded a video clip while reading five lists of 25 monosyllabic Korean words against eight-talker babble noise, using a lapel microphone (BY-WMA4 PRO K3, BOYA, Shenzhen, Hong Kong) and a cellphone camera (iPhone 12 mini, Apple, Cupertino, CA, USA), once without wearing a mask and again with the Korean filter 94% (KF94) mask. The KF94 mask has been certified by the Korean Food and Drug Administration and is made of four layers of unwoven material with an ultra-electrostatic filter. The dustproof effect is similar to those of filtering facepiece 2 on European standards and N95 on American standards. The recorded video clip was provided *via* a monitor and a loudspeaker placed 1 m in front of the subject in a soundproof booth under the no-mask–AV and mask–AV conditions, whereas only sound was provided under the no-mask–AO conditions. Before the experiment, we showed the subjects a silent video, with the monitor at a distance of 1 m, and confirmed that the speaker’s mouth shape was clearly visible. To minimize redundancy cues and sematic cues in speech perception and to force the listener to focus on the speech cues themselves, we used monosyllabic words. Five lists of 25 monosyllabic words were used to test five noise levels (−16, −12, −8, −4, and 0 dB SNR), and the order of the words in the lists was randomly assigned for each of the no-mask–AO, mask–AV, and no-mask–AV conditions. The phonetic balance, equal range of the phonetic composition of speech, words in common usage, and the words’ familiarity was considered when the word lists were developed. The equivalent average difficulty and phoneme composition of the lists were verified. The subjects were asked to repeat the words verbally while ignoring the noise and the number of correct responses was measured for a total of 25 words. The tests were performed in the following order: no-mask–AO, mask–AV, and no-mask–AV conditions. Under each condition, the order was −16, −12, −8, −4, and 0 dB SNR. For the hearing-impaired subjects, the tests were performed first without hearing aids, and then performed again with hearing aids. Speech perception was measured by fixing the monosyllabic words at 65 dB SPL when calibrated at the listeners head position 1 m away from the loudspeaker and changing the speech-shaped noise to five scales of −16, −12, −8, −4, and 0 dB SNR.

### Acoustic analysis

The same female speaker who made the video clip recorded standard Korean phrases with similar proportions of consonants and vowels using a lapel microphone (BY-WMA4 PRO K3, BOYA, Shenzhen, Hong Kong) in a soundproof booth. With and without the KF94 mask, and the long-term average speech spectrum of the recorded phrase was analyzed with the Computerized Speech Lab (CSL model 4500 b, KayPENTAX Elemetrics Corporation, Lincoln Park, NJ, USA).

### Statistical analysis

A 2 × 3 × 5 mixed repeated-measures analysis of covariance (ANCOVA), controlled for age (covariate), was used to analyze the effects of the hearing status (normal hearing group or hearing-impaired group), the experimental conditions (no-mask–AO, mask–AV, or no-mask–AV), and the noise level (−16, −12, −8, −4, or 0 dB SNR) on speech perception in noise. To compare the effects of masks on speech perception when hearing aids were worn in the hearing-impaired group, a 2 × 3 × 5 (two amplifications, three experimental conditions, and five noise levels) pure repeated-measures analysis of variance (ANOVA) was used. In the *post-hoc* test, the significance levels after Bonferroni’s correction of the *p*-values were deemed to be 0.003 (0.05/15) or 0.01 (0.05/5) for multiple comparisons. All statistics were performed using IBM SPSS software (ver. 25.0; IBM Corp, Armonk, NY, USA).

## Results

### Spectral analysis

**FIGURE 2:**
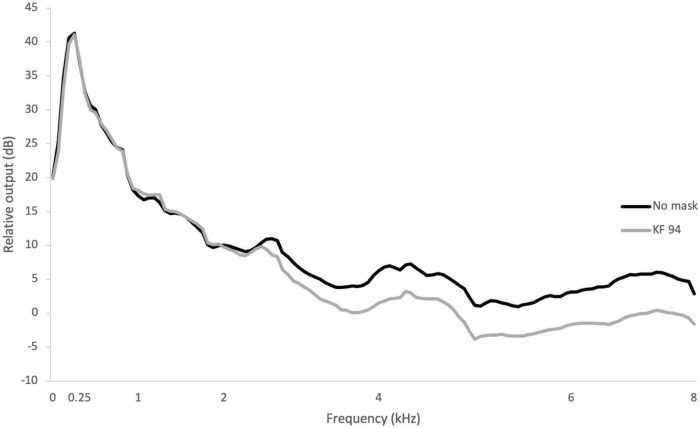
Long-term speech spectra under the no-mask and mask conditions.

The long-term average spectrum of the Korean-standardized phrase with the KF94 mask demonstrated an average attenuation of 4.0 (*SD* = 1.5) dB at ≥2 kHz relative to the no-mask condition. The maximum reduction in sound level was around 6.2 dB at 7 kHz (Figure 2).

### Effect of mask wearing on speech perception in noise; comparison of the normal-hearing and hearing-impaired groups (without hearing aids)

**FIGURE 3:**
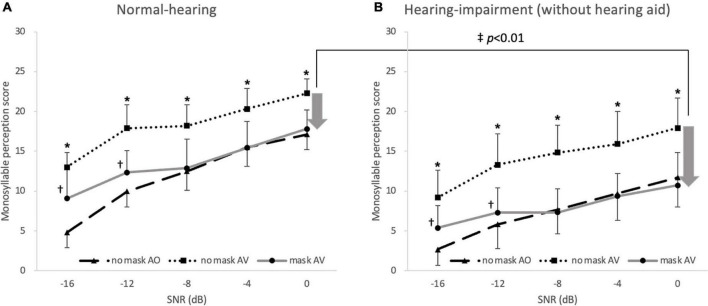
Monosyllabic speech perception in noise under no-mask–auditory–verbal (AV; filled squares), mask–AV; filled circles, and no-mask–auditory only (AO; filled triangles) conditions. In the **(A)** normal-hearing and **(B)** hearing-impaired groups, the speech perception score was lower under the no-mask–AO and mask–AV conditions than under the no-mask–AV conditions at all noise levels (*p < 0.003 vs. no-mask–AV, Bonferroni’s corrected α = 0.05/15 = 0.003), and the speech perception score was better under mask–AV conditions than under no-mask–AO conditions at –16 and –12 dB SNR in the normal-hearing and hearing-impaired groups (^†^*p* < 0.003, Bonferroni’s corrected α = 0.05/15 = 0.003). The degree of deterioration in speech perception caused by the mask (no-mask–AV minus mask–AV) was significantly greater at 0 dB SNR only in the hearing-impaired group (^‡^*p* < 0.01, Bonferroni’s corrected α = 0.05/5 = 0.01). The arrow indicates the degree of deterioration in speech perception and the error bar indicates SD.

A mixed three-way repeated-measures ANCOVA (two hearing status × three experimental conditions × five SNRs), controlled for age (covariate), showed significant main effects for hearing status [*F*_(1,__47)_ = 38.85, *p* < 0.001], the experimental condition [*F*_(1.64,76.96)_ = 9.30, *p* = 0.01], and SNR [*F*_(4, 188)_ = 16.19, *p* < 0.001]. The covariate age was significantly related to monosyllabic perception [*F*_(1,__47)_ = 6.47, *p* = 0.014]. The planned contrasts revealed that the hearing-impaired group (*p* < 0.001, 95% CI [−5.13, −2.63]) had significantly worse monosyllabic perception than the normal-hearing group. There was no significant interaction among the three factors (all *p* > 0.05), but the interaction between hearing status and SNR tended toward significance (*p* = 0.051). In the *post-hoc* test, speech perception was lower under both the no-mask–AO and mask–AV conditions than under the no-mask–AV conditions in both the normal-hearing and hearing-impaired groups (without hearing aids) at all noise level (*p* < 0.003, accepted α = 0.003). In both the normal-hearing group and hearing-impaired group (without hearing aids), the mask–AV conditions produced better speech perception than the no-mask–AO conditions at −16 and −12 dB SNR [*t* (25) = −3.81 and *t* (25) = −5.28 in the normal-hearing group, *t* (23) = −5.94 and *t* (23) = −2.74 in the hearing-impaired group, *p* < 0.003, accepted α = 0.003], but there was no difference at −8, −4, or 0 dB SNR. When the degree of deterioration in speech perception caused by the mask (no-mask–AV minus mask–AV) was compared between two groups at each noise level, the difference was only significantly greater in the hearing-impaired group than in the normal-hearing group at 0 dB SNR [*M* = 7.2 and *SD* = 3.7 vs. *M* = 5.2 and *SD* = 2.2, *t* (36.54) = −2.23, *p* = 0.032, accepted α = 0.01; Figure 3].

### Effect of mask wearing on the benefit of hearing aids

**FIGURE 4:**
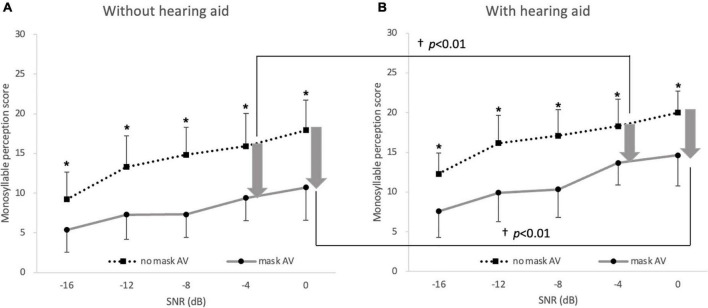
Deterioration in monosyllabic speech perception in noise caused by mask. Speech perception was lower under the mask–AV conditions (filled circles) than under the no-mask–AV (filled squares) conditions **(A)** without hearing aids and **(B)** with hearing aids at all noise levels (*p < 0.003, accepted α = 0.003). The degree of deterioration in speech perception caused by a mask was significantly lower with hearing aids than without hearing aids at 0 and –4 dB SNR (^†^*p* < 0.01, Bonferroni’s corrected α = 0.05/5 = 0.01). The arrow indicates the degree of deterioration in speech perception and the error bar indicates SD.

In the hearing-impaired group, a pure three-way repeated-measures ANOVA (two amplification conditions [with or without hearing aids] × three experimental conditions × five SNRs) showed significant main effects for hearing aids [*F*_(1,__23)_ = 66.70, *p* < 0.001], the experimental condition [*F*_(2,__46)_ = 280.29, *p* < 0.001], and SNR [*F*_(4, 92)_ = 161.52, *p* < 0.001]. There were significant interactions between the condition and SNR [*F*_(8, 184)_ = 8.14, *p* < 0.001] and among the three factors [*F*_(8, 184)_ = 2.24, *p* = 0.027], but no other significant interaction was observed (all *p* > 0.05). When the effects of a mask on speech perception with and without hearing aids were compared in the hearing-impaired group, the degree of deterioration in speech perception caused by the mask (no-mask–AV minus mask–AV) was significantly reduced by the hearing aids compared with the degree of deterioration without hearing aids at 0 dB SNR and −4 dB SNR [*t* (23) = 2.41 and *t* (23) = 3.95, *p* < 0.01, accepted α = 0.01; Figure 4].

**FIGURE 5:**
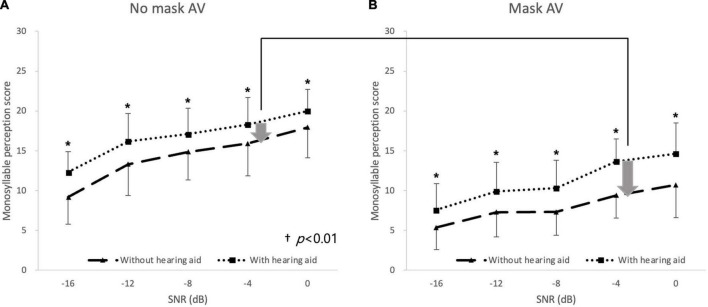
Monosyllabic speech perception in noise with and without hearing aids. In the hearing-impaired group, speech perception was better with hearing aids than without hearing aid at all noise levels under **(A)** no-mask–auditory–verbal (AV) and **(B)** mask–AV conditions (*p < 0.003, Bonferroni’s corrected α = 0.05/15 = 0.003), and the difference in speech perception at –4 dB SNR depending on wearing hearing aids was greater under the mask–AV conditions than under the no-mask–AV conditions (^†^*p* < 0.01, Bonferroni’s corrected α = 0.05/5 = 0.01).

In the hearing-impaired group, hearing aids significantly improved speech perception relative to that without hearing aids at all noise levels under both the no-mask–AV conditions and mask–AV conditions (*p* < 0.003, accepted α = 0.003; Figure 5). The gain conferred by hearing aids (aided minus unaided) on speech perception at −4 dB SNR among 5 SNRs was greater under the mask–AV conditions than under the no-mask–AV conditions [*M* = 7.2 and *SD* = 3.7 vs. *M* = 5.2 and *SD* = 2.2, *t* (23) = −3.25, *p* < 0.01, accepted α = 0.01].

## Discussion

Most kinds of masks attenuate frequencies above 1–2 kHz (Corey et al., 2020; Magee et al., 2020; Rahne et al., 2021), although there is a difference in the degree of attenuation depending on the type of mask. Surgical masks cause the smallest departures from the original speech spectrum and transparent window masks cause the greatest attenuation (Corey et al., 2020). The average attenuation level of the KF94 mask used in this study is similar to that of N95 masks, which have equivalent dustproof effects, at 4–5 dB. The shape of the spectrum of masked speech for N95 and KF94 masks is also similar.

In this study, we confirmed that speech perception was better under the no-mask–AV conditions than under the no-mask–AO conditions at all noise levels, regardless of hearing status. The multisensory integration of auditory and visual stimuli improves speech recognition in noise compared with that under unimodal stimuli (Giard and Peronnet, 1999; Liu et al., 2013). Neuroimaging data have also shown that a stronger response occurs in the auditory cortex when auditory and visual stimuli are received together than when an auditory stimulus is received alone (Kayser et al., 2007). Several studies have demonstrated that greater noise is associated with a greater gap in perception between the AV and AO conditions (Sumby and Pollack, 1954). However, we detected no such trend in the setting of the present study. Similarly, because a mask blocks the visual information derived from the speaker’s mouth shape, the poorer speech perception under mask–AV conditions than under no-mask–AV conditions was expected. Several studies have shown that a mask reduces speech perception more strongly against a high level of noise than against a low level of noise in normal- hearing individuals (+ 3 vs. + 13 dB SNR, Toscano and Toscano, 2021; 0 dB vs. + 5 dB SNR, Smiljanic et al., 2021). In contrast, in the normal-hearing group of the present study, no difference in degree of deterioration in speech perception was detected at different levels of background noise, which may be attributable to the relatively high noise levels used in this experiment. Moreover, because AV speech perception can be influenced by cultural factors (Sekiyama, 1997), the differences between the results of previous studies (mostly conducted in the West) and those of this study may be attributable to cultural factors. The adverse effect of the mask on speech perception arises not only from the blocking of the visual information provided by the speaker’s mouth shape, but also from the distortion of sound. Therefore, we anticipated worse speech perception under the mask–AV conditions than under the no-mask–AO conditions. On the contrary, speech perception was rather better under the mask–AV conditions than under the no-mask–AO conditions at relatively high noise levels (−16 and −12 dB SNR). This means that in severely degraded listening conditions, the visual contribution of the speaker’s partial facial expression, excluding the motion of the mouth, can overwhelm the sound distortion effect of the mask. Speech-reading is a multidimensional skill that draws on not only the motion of the mouth and lips but also the expression of the other parts of the face (Lyxell and Rönnberg, 1989). Although many studies have compared speech perception under no-mask and masked conditions, few studies have evaluated the difference between no-mask–AO and mask–AV conditions. Atcherson et al. reported that unlike participants with normal hearing, those with severe hearing impairment had better speech perception when they listened to speech under no-mask–AV conditions or with a transparent mask that provided visual cues than when a mask covered the movements of the mouth (Atcherson et al., 2017).

We hypothesized that the adverse effects of a mask on speech perception is greater in hearing-impaired individuals than in normal-hearing individuals because hearing-impaired people have more difficulty when sound is distorted by a mask and are also more dependent on lip reading than are normal-hearing people. The hypothesis was true only at the lowest noise level of 0 dB SNR. Our data are largely consistent with those of previous studies (Thibodeau et al., 2021; Alkharabsheh et al., 2022), which were performed under conditions of a relatively robust signal (Thibodeau et al., 2021; Alkharabsheh et al., 2022), although the experimental conditions of our study involved more intense noise environments than those studies. For this reason, in this study, when the noise level exceeded −4 dB SNR, the effect of the mask on speech perception did not differ according to hearing status. Speech perception may vary depending on the type of mask used. In another study, the presence of a surgical mask had no detrimental effect on speech perception in either the normal-hearing or hearing-impaired group (Mendel et al., 2008). The contribution of visual cues to speech perception according to hearing status was evaluated, but the results of previous research varied according to the experimental setting. Several studies showed a positive correlation between the degree of hearing loss and AV enhancement in speech perception (Altieri and Hudock, 2014; Puschmann et al., 2019), but others detected no difference in the benefits of visual cues according to the degree of hearing loss (Tye-Murray et al., 2007; Rosemann and Thiel, 2018). Taken together, these data indicate that masks tend to have a greater adverse effect on hearing-impaired people than on normal-hearing people, but this may vary with the type of mask and the background noise level.

To the best of our knowledge, this is the first study to evaluate how masks affect the speech perception of individuals with hearing aids, based on a within-subject comparison of the mask effect with or without hearing aids. We showed that hearing aids still improved speech perception in a mask-wearing environment, and that the gain in speech perception conferred by hearing aids was greater under mask–AV conditions than under no-mask–AV conditions at only one of the five noise levels tested. The adverse effect of the mask on speech perception was significantly reduced by wearing hearing aids at noise levels of 0 and −4 dB, implying that the hearing aids partly offset the mask effect at relatively low noise levels. However, at relatively high noise levels, the hearing aids did not offset the mask effect, which may be related to the technical limitations of the hearing aids themselves. Hearing aid technologies that improve the SNR, such as directional microphones, would have been ineffective in this research context where the signals and noise were provided simultaneously in front of the listener.

A limitation of this study was that the mean age of the hearing-impaired group (*M* = 69.5, *SD* = 8.6 years) was significantly higher than that of the normal-hearing group (*M* = 57.9, *SD* = 11.1 years). However, the effect of this age difference on the outcomes is presumed to be minimal. A previous large survey of 1,700 people showed that the age of the listener did not affect the degree of communication disturbance caused by masks (Helfer et al., 2021). Moreover, ANCOVA was used to control for the age difference between the two groups. Another limitation was the use of a single type of face mask. Different types of mask, such as surgical masks, transparent masks, and cloth masks, could produce different results. The use of monosyllabic words for the speech perception test could be a limitation of this study because monosyllabic words do not fully reflect real-word experience. The effects of co-articulation for monosyllabic words are not the same as sentences, and monosyllabic words may not adequately allow normal speech-reading, in which the motion of the mouth and face is considered.

In conclusion, the perception of masked speech by hearing-impaired individuals was more adversely affected against relatively low background noise than was that of normal-hearing individuals. Hearing aids still improved speech perception in a mask-wearing environment, and hearing aids partly offset the mask effect at relatively low noise levels.

## Data availability statement

The original contributions presented in this study are included in the article, further inquiries can be directed to the corresponding author.

## Ethics statement

The studies involving human participants were reviewed and approved by the Institutional Review Board of Nowon Eulji Medical Center. The patients/participants provided their written informed consent to participate in this study.

## Author contributions

HS: conceptualization, funding acquisition, and critical review. HS, Y-HA, and JC: experiment design. HC: experiment performance. JC: data preparation and analysis. HC, DK, and JP: methodology. JC and HS: writing manuscript. All authors have read and agreed to the published version of the manuscript.
